# Supplementary figures and images for: Structural and functional properties of a magnesium transporter of the SLC11/NRAMP family
Source: eLife. 2022 Jan 10;11:e74589. doi: 10.7554/eLife.74589 (PMC8806188; doi:10.7554/eLife.74589)

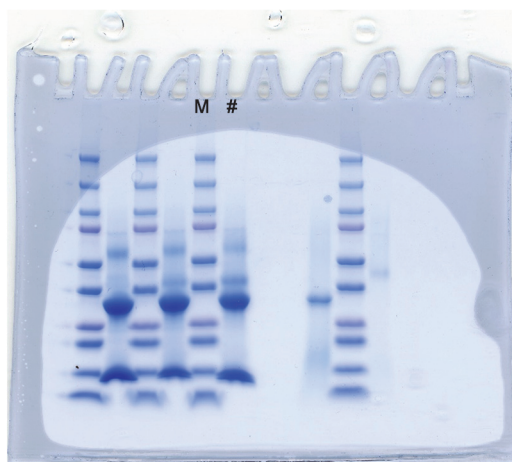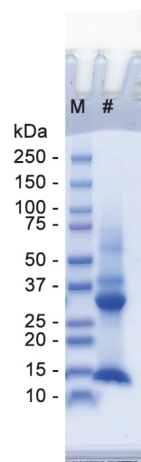

Supplement: Source data 1. [file elife-74589-supp1.zip › source data/Figure4_supplement1D_gel.pdf]
